# Antiplasmodial Activity Assay of 3-Chloro-4-(4-chlorophenoxy) Aniline Combinations with Artesunate or Chloroquine *In Vitro* and in a Mouse Model

**DOI:** 10.1155/2019/5153482

**Published:** 2019-10-17

**Authors:** Martin Wekesa Sifuna, Milka Wambui, Joseph Kang'ethe Nganga, Daniel Wainaina Kariuki, Francis Thuo Kimani, Francis Wamakima Muregi

**Affiliations:** ^1^Biochemistry Department, Jomo Kenyatta University of Agriculture and Technology, P.O. Box 62000, Nairobi, Kenya; ^2^Centre for Biotechnology Research and Development, Kenya Medical Research Institute (KEMRI), P.O. Box 54840, Nairobi, Kenya; ^3^Department of Biological Sciences, Mount Kenya University, P.O. Box 342, Thika, Kenya

## Abstract

Malaria is the eighth highest contributor to global disease burden with 212 million cases and 429,000 deaths reported in 2015. There is an urgent need to develop multiple target drug to curb growing resistance by *Plasmodia* due to use of single target drugs and lack of vaccines. Based on a previous study, 3-chloro-4-(4-chlorophenoxy) aniline (ANI) inhibits *Plasmodia* enoyl acyl carrier protein reductase. This study aimed at evaluating the antiplasmodial activity of ANI combinations with artesunate (AS) or chloroquine (CQ) against *P. falciparum in vitro* based on the semiautomated microdilution assay and *P. berghei in vivo* based on Peters' 4-day test. Data were analysed by linear regression using version 5.5 of Statistica, 2000. From the results, on the one hand, a combination of 1.1 ng/ml AS and 3.3 *μ*g/ml of ANI inhibited 50% growth of W_2_, while a combination of 0.8 ng/ml of AS and 2.6 *μ*g/ml of ANI inhibited 50% growth of 3D_7_. On the other hand, a combination of 22 ng/ml CQ and 3.7 *μ*g/ml of ANI inhibited 50% growth of W_2_, while a combination of 4.6 ng/ml CQ and 3.1 *μ*g/ml of ANI inhibited 50% growth of 3D_7_. In *in vivo* assays, a combination of ED_50_ concentrations of AS and ANI cleared all parasites, while 1/2 and 1/4 ED_50_ combinations inhibited 67.0% and 35.4% parasite growth, respectively. ED_50_ combinations of CQ and ANI inhibited 81.0% growth of parasites, while 1/2 and 1/4 ED_50_ combinations inhibited 27.3% and 10.2% parasite growth. Assuming a linear relationship between percentage chemosuppression and combination ratios, only 0.88 mg/kg of AS combined with 1.68 mg/kg of ANI or 1.78 mg/kg of CQ with 3.15 mg/kg of ANI inhibited 50% parasite growth *in vivo*. ANI combinations with AS or CQ are thus potential antimalarial drug combinations if their clinical efficacy and safety are ascertained.

## 1. Introduction

Malaria remains a major human parasitic disease despite the substantial progress made in the fight against it since 2000 [1]. At the beginning of 2016, malaria was considered to be endemic in only 91 countries and territories, down from 108 in 2000 which was attributed to wide-scale deployment of control interventions which reduced incidence by 41% and mortality rates by 62%. Updated estimates between 2000 and 2015 indicate that 212 million malaria cases occurred globally in 2015, causing 429 000 deaths, especially of expectant mothers and children under 5 years [1].

The plasmodium is transmitted by the female Anopheles mosquito which releases sporozoites into the bloodstream during a blood meal. The parasites evade the host immune system by sequestering inside the red blood cells (RBCs) and the hepatocytes [2]. Usually, the infected RBCs adhere to the walls of blood capillaries evading destruction in the spleen [2]. The sequestration of infected RBCs is highly associated with placental and cerebral malaria [3]. Headache, shivering, and joint pain may begin to manifest 8 to 25 days after infection [4]. Draining of stagnant water, indoor residual spraying (IRS), and use of insecticide-treated nets (ITNs) are the most cost-effective parasite vector control measures [5]. ITNs offer over 70% protection, and between 2000 and 2008, they saved the lives of about 250,000 babies in sub-Saharan Africa, prompting WHO to recommend distribution of affordable ITNs to women in early pregnancy [6].

With challenges in vector control and vaccine development, chemotherapy remains as the mainstay of the malaria control strategy [1]. Quinine was for a long time the drug of choice for management of malaria. Quinine has encountered resistance by the parasite prompting changes to other therapies [7]. Chloroquine (CQ) was a serendipitous drug against *Plasmodia* used for several decades after quinine, but its use was also halted by development of resistance by the parasite [8]. As a result, artemisinin-based combination therapy (ACT) was adopted as the first-line regimen against falciparum malaria [1]. When the parasite ingests haemoglobin, it releases the heme which activates the artemisinin derivatives such artesunate (AS) generating reactive oxygen radicals that destroy the parasite membranes [9]. AS also inhibits a membrane glutathione s-transferase enzyme that metabolizes the products of lipid peroxidation. Accumulation of lipid peroxides thus kills the parasites [10]. Being a combination of more than one drug, ACT was quite effective against the malaria parasites and AS, one of the drugs on the WHO list of essential medicines, is the most appropriate drug against severe and complicated malaria [11]. The number of course treatments using ACTs had increased from 11 million in 2005 to 337 million by 2014 in an effort to curb growing resistance by plasmodia. Despite continuous effort against malaria, some *Plasmodia* strains in malaria-endemic regions like Cambodia are developing resistance to the ACTs.

At present, there is still an urgent need to develop new drug combinations early as parasite resistance against present drugs seems to outstrip the rate of drug development which is usually a long process. In a spirited search for a remedy to resistance menace, *in silico* studies on 3-chloro-4-(4-chlorophenoxy) aniline (ANI) have been attempted based on the structural similarity between ANI and triclosan. Triclosan is an antibacterial agent used in tooth paste to inhibit an enoyl-acyl carrier protein reductase enzyme which catalyses the reduction of an enoyl-ACP to an acyl-ACP in the type II (FASII) fatty acid biosynthesis pathway [12]. The FASII pathway is also vital for the development of liver stages of plasmodia [12]. Although the parasite scavenges for fatty acids, destruction of the FASII pathway affects oocyst maturation and sporozoite formation since the pathway supplies specific fatty acids essential for lipid and membrane biogenesis [13]. The FASII pathway is therefore a profound drug target in malaria chemotherapy and hence the proposal to use ANI against plasmodium. Based on this fact, we assayed the antiplasmodial activity of ANI combinations with AS or CQ *in vitro* against laboratory-adapted *P. falciparum* isolates (CQ sensitive, 3D_7_; CQ resistant, W_2_) and *in vivo* against *P. berghei.* The main objective was to elucidate antiplasmodial activity of ANI and the nature of the drug-drug interactions as either additive, synergistic, or antagonistic and hence the feasibility of using the combinations as an additional ACT to curb malaria.

## 2. Materials and Methods

### 2.1. Parasite Cultures and Drug Preparation

This study was done in Kenya Medical Research Institute (KEMRI). For *in vitro* studies, two strains of *P. falciparum*, CQ-sensitive (3D_7_), and CQ-resistant (W_2_), cryopreserved in the KEMRI malaria laboratory were used, while *P. berghei ANKA* maintained in mice was used for *in vivo* studies. The drugs were procured from Sigma-Aldrich®, prepared, and used freshly. CQ was prepared by dissolving 5 mg in distilled water and then diluted with RPMI to a starting concentration of 1000 ng/ml, while 5 mg of AS and 5 mg ANI was separately dissolved in 0.5 ml of DMSO and diluted with RPMI to starting concentrations of 100 ng/ml and 1000 *μ*g/ml, respectively.

### 2.2. *In Vitro* Assays for AS, CQ, and ANI

*In vitro* parasite sensitivities to individual drugs were assessed as described in [14]. In brief, 50 *μ*l of the highest concentration of AS was added to all wells in row B of the 96-well flat-bottomed microtitre plate and then serially diluted two-fold downwards. A 200 *μ*l suspension of *P. falciparum* parasitized RBC was added to each well forming a 1% haematocrit. Column A was the positive and negative control well with no drug. Raw A constituted the control setup. The positive controls had 1% suspension of infected RBCs in the complete medium, while the negative control wells contained the unparasitized RBC suspension. This protocol was repeated for CQ and ANI. The plates were cultured at 37°C under microaerobic environment (91% N_2_, 6% CO_2_, and 3% O_2_) for 48 hours after which 25 *μ*l of radiolabelled hypoxanthine was added and incubated for 18 more hours. The plates were then frozen at −70°C overnight and RBC lysed by thawing to release the parasites which were harvested onto glass fibre filters and dried, and radioactivity was measured by liquid scintillation in counts per minute (cpm). The concentrations causing 50% inhibition of radioisotope incorporation (IC_50_) which signify presence of life parasites were determined by interpolation after logarithmic transformation of both concentration and the cpm values using the formula:

(1) $$\begin{array}{c} {\text{IC}}_{50}=\text{anti}\log\left(\log\;X_{1}+\left[\frac{\left(\log\;Y_{50}-\log\;Y_{1}\right)\left(\log\;X_{2}-\log\;X_{1}\right)}{\left(\log\;Y_{2}-\log\;Y_{1}\right)}\right]\right), \end{array}$$

where *Y*_50_ is the midpoint; *X*_1_ and *X*_2_ are lower and upper concentrations, respectively; and *Y*_1_ and *Y*_2_ are the corresponding cpm values for the data points above and below the cpm midpoints [15].

### 2.3. *In Vitro* Assays of Drug Combinations

Based on the individual IC_50_ obtained in Section 2.2, the drug-drug interaction assays were done as described by Canfield et al. [16]. In brief, fixed-ratio combinations of drug A and drug B (9 : 1 to 1 : 9) were prepared in a manner that the IC_50_ for individual drugs falls in the 4^th^ serial dilution. Each fixed-ratio combination of desired concentrations was prepared, and 50 *μ*l of the starting concentration was added to all wells in row B and then serially diluted two-fold. A 200 *μ*l suspension of *P. falciparum* parasitized erythrocytes was added to each well forming a 1% haematocrit. With wells in column A as controls, the culturing and parasite harvesting and counting were done, as aforementioned in Section 2.2, and the respective inhibitory concentrations were determined as described by Sixsmith. Fractional inhibitory concentrations (FIC) of the drugs were calculated as follows: FIC of drug A = [IC_50_ of drug A in combination/IC_50_ of drug A alone] and FIC of drug B = [IC_50_ of drug B in combination/IC_50_ of drug B alone], from which sum FIC_50_ was derived. A sum of FIC value <1.0 denotes synergy, 1.0 denotes additive effect, while a value >1.0 denotes antagonism [17].

### 2.4. Cytotoxicity Assays of Drug Combinations

The present study focused on the toxicity assay for drug combinations with the aim of developing drugs with multiple targets on parasite to reduce resistance development. Therefore, the cytotoxicity assays of ANI combinations with AS or CQ on Vero cell lines were determined using the 3-(4,5-dimethylthiazol-2-yl)-2,5-diphenyltetra-zolium bromide (MTT) cell viability assay [18]. In brief, Vero E6 cells were cultured in minimum essential media supplemented with 10% foetal bovine serum fortified with 1% streptozomycine and incubated at 37°C and 5% CO_2_. The confluent cultures were trypsinized, and concentration was determined by the trypan blue exclusion assay. Of these, 100 *μ*l containing 2.0 × 10^4^ cells were transferred into each experimental well in the 96-well culture plate and incubated for 24 hrs at 37°C and 5% CO_2_ to form about 80–100% monolayer/confluent. AS (50 *μ*l) was added into the first 2 wells to form the highest drug concentration of 1000 *μ*g/ml. For the combined drugs, 25 *μ*l of AS was added to 25 *μ*l of ANI and added into the wells containing 2.0 × 10^4^ cells in 100 *μ*l culture media for the highest drug concentration of 1000 *μ*g/ml (consisting 900 *μ*g/ml of the ANI and 100 *μ*g/ml of AS). The protocol was repeated for the CQ-ANI combination using the highest combination drug concentration of 1000 *μ*g/ml (consisting 900 *μ*g/ml of the ANI and 100 *μ*g/ml of CQ). These were then serially diluted downwards and incubated in a humidified incubator at 37°C. At 48 hours postincubation, an MTT dye was added to the wells and allowed to incubate at 37°C for further 3 hours, and the colorimetric reaction stopped by the addition of 100 *μ*l of DMSO. The spectrometric absorbance of each well was measured at 450 nm using scanning multiwell spectrometer (Multiskan EX Lab systems). The data acquired were transferred into the Microsoft excel software (2007) to determine the concentration causing 50% visible alteration of intact cells (IC_50_ or CC_50_) as follows: % cell viability (CC_50_) = [OD_sample 562_ − OD_620_/OD_control562_ − OD_620_] × 100 [19].

### 2.5. *In Vivo* Assays for AS, CQ, and ANI

The four-day suppressive test was adapted for *in vivo* tests [18] In brief, 65 Swiss albino mice of 20 ± 2 g average weight were infected intraperitoneally (ip) with 0.2 ml inoculum containing about 10^5^*P. berghei* ANKA parasitized RBCs from donor mice on day 0.

The mice were first randomized into 13 groups of 5 mice each and then into 4 groups for each drug with one negative control group and fed on pellets and watered ad libitum. Three hours postinfection (pi), the drugs are orally administered, as indicated in Table 1 and repeated once daily for 3 days with the negative control mice receiving water containing the same percentage of DMSO. On day 4 pi, percentage chemosuppression was calculated from Giemsa-stained thin tail-blood smears from mice using the following formula: [(A − B)/A] × 100 = % chemosuppression, where A is the mean parasitaemia in the negative control group and B is the mean parasitaemia in test groups. Dosages killing 50% of parasite (ED_50_) for each drug were determined by linear regression using version 5.5 of Statistica, 2000.

### 2.6. *In Vivo* Assays for Drug Combinations

*In vivo* drug interactions were assessed, as described by Ishih et al. [20]. In brief, 3 fixed-ratio drug doses of ANI combinations with AS or CQ were freshly prepared. Thirty-five Swiss albino mice (20 ± 2 g) were infected ip with 0.2 ml blood containing 10^5^*P. berghei* ANKA parasitized RBC and randomized into 7 groups of 5 mice each. AS and CQ were assigned 3 groups each. Three hours postinfection, one group from the drug groups received a combination of full doses, i.e., the individual ED_50_ quantities of each drug, the other group half the individual ED_50_ quantities of each drug, and the last group received a quarter of the ED_50_ quantities of each drug. The negative control mice received water containing the same percentage of DMSO. Drugs were orally administered once daily for 3 days. On day 4 pi, percentage chemosuppression was calculated from Giemsa-stained thin tail-blood smears and their ED_50_ was determined by linear regression using version 5.5 of Statistica, 2000. Student's *t*-test was used to determine statistical significance at 95% confidence level.

### 2.7. Data Analysis

The top-count microplate scintillation and luminescence counter provided the *in vitro* results as cpm per minute expressed as percentage of the controls. IC_50_ was estimated by linear interpolation [21]. The data were then analysed in Microsoft Excel, and IC_50_ concentrations of the drugs were obtained by logarithm-transformed concentration values [22]. The IC_50_ and standard deviation values were calculated from duplicate values for all drugs tested. For *in vivo* assays, percentage parasitaemia was recorded in Excel (Microsoft) and expressed as the mean ± standard error of mean. ED_50_ was determined by linear regression using version 5.5 of Statistica 2000. Statistical analysis of the data was done using Student's *t*-test with a significance level set at *p* < 0.05.

## 3. Results

### 3.1. *In Vitro* IC_50_ Values for AS, CQ, and ANI and Their Combinations

Table 2 illustrates the IC_50_ values of AS, CQ, and ANI and their respective combinations for the two parasite strains, 3D_7_ and W_2_. The parasite was less responsive to the ANI as compared to CQ and AS which was the most active against the *Plasmodia*.

In combinations, only 0.8 ng/ml of AS combined with 2.6 *μ*g/ml of ANI as opposed to 1.6 ng/ml of AS and 7.12 *μ*g/ml of ANI individually administered attained 50% 3D_7_ growth inhibition. Similarly, only 1.1 ng/ml of AS combined with 3.3 *μ*g/ml of ANI inhibited 50% growth of W_2_ as compared to 2.4 ng/ml of AS and 8.75 *μ*g/ml of ANI individually required similar inhibition. At all combinations of ANI with either AS or CQ, the drug interactions demonstrated synergy with sum FIC values <1, as shown in Table 3.

### 3.2. Cytotoxicity Assays for Drug Combinations

The selective index of the combination drugs was determined by finding the ratio between IC_50_ of the drug against Vero E6 cells and against parasites. The 50% cytotoxic level of ANI combination with AS was 35.43 *μ*g/ml with a selective index of 13.6 while that of ANI combination with CQ was 41.13 *μ*g/ml with a selective index of 13.3, as shown in Table 4.

### 3.3. *In Vivo* Inhibitory Properties of AS, CQ, and ANI and Their Combinations

Of the three drugs tested *in vivo*, AS was the most active drug with an ED_50_ of 2.2 mg/kg body weight, while CQ had an ED_50_ of 2.38 mg/kg body weight. The ANI had an ED_50_ of 4.18 mg/kg and ED_90_ of 9.1 mg/kg body weight, as shown in Table 5.

The ANI combination with AS was more synergistic both at ED_50_ and lower combination values, while ANI combination with CQ showed trends towards synergy at higher combination concentrations alone. ED_50_ combination of AS and ANI was able to clear the parasites, while half ED_50_ combinations still produced an inhibition higher than 50%, a show of synergy. On the contrary, ED_50_ combination of CQ and ANI had 81% growth inhibition on the parasite. The drug combinations needed to attain 50% parasite growth suppression was 0.88 mg/kg of AS combined with 1.68 mg/kg of ANI or 1.78 mg/kg of CQ combined with 3.15 mg/kg of ANI.

## 4. Discussion

Evolution of drug-resistant parasites to antimalarial drugs including ACTs is a major hindrance to malaria control, and therefore, effective management of malaria will only be realized through development of new therapies [22]. Constant monitoring of developing resistance and development of new therapies, needed approximately every ten years, is of utmost importance so as to keep malaria control efficacious [23]. Coformulation of antimalarial drugs with multiple targets optimizes efficacy due to synergistic effects of one drug on the other, and delaying the development of resistance is the current strategy in development of malaria therapies. Drug combination therapy also reduces chances of toxicity, increasing safety and drug tolerability, as well as increasing the efficacy of member drugs [24].

Of the three drugs under the *in vitro* study, AS was the most active with IC_50_ values of 1.6 ng/ml against 3D_7_ and 2.4 ng/ml against W_2_, while CQ had an IC_50_ value of 10.9 ng/ml against 3D_7_ and 48 ng/ml against W_2_. The ANI was less effective *in vitro* as compared to either AS or CQ with IC_50_ values of 7.12 *μ*g/ml against 3D_7_ and 8.75 *μ*g/ml against W_2_ (Table 2). ANI's different mechanisms of action make it an ideal partner drug to the present antimalarial drugs. Indeed, a combination of ANI with AS or CQ exhibited increased parasite clearance (Table 3), as compared to administration of individual administration drugs. An AS combination with ANI was more potent both *in vitro* and *in vivo* cases with only 1.1 ng/ml of AS (IC_50_; 2.4 ng/ml), and 3.3 *μ*g/ml of ANI (IC_50_; 8.75 *μ*g/ml) was needed to attain 50% inhibition of *P. falciparum*, W_2_ with a sum FIC of 0.82 on W_2_, and 0.86 on 3D_7_ (Table 3). Similar synergistic effects were also demonstrated between the CQ combination with ANI where only 22 ng/ml of CQ (IC_50_; 48 ng/ml) and 3.7 *μ*g/ml of ANI (IC_50_; 8.75 *μ*g/ml) were required to produce 50% inhibition of growth of W_2_, and only 4.6 ng/ml of CQ combined with 3.1 *μ*g/ml of ANI produced 50% inhibition of growth of 3D_7_ with a sum FIC of 0.87 on W_2_ and 0.85 on 3D_7_ (Table 3). These findings thus point at the potential of the ANI combinations with AS or CQ as a possible remedy to the growing parasite resistance to the present antimalarial therapies.

Acute cytotoxicity and toxicity assays are paramount in predicting the possible risks of the formulated drugs in humans exposed to fatal doses, enabling anticipation of the nature of an acute response by body systems [25]. The IC_50_ of AS was 62.32 ng/ml, while its combination with ANI had an IC_50_ of 35.43 *μ*g/ml with a selective index of 13.6. CQ combination with ANI had an IC_50_ of 41.13 *μ*g/ml with a selective index of 13.3. With the selective index of over 10 times the therapeutic levels (Table 4), these drug combinations are safe, considering the low amounts of drug combinations that course a remarkable parasitaemia suppression.

At 5 mg/kg of ANI, AS, and CQ in individual *in vivo* studies, ANI demonstrated a remarkable parasite chemosuppression capacity (75.3%) relative to the controls (*p* < 0.05), with an ED_50_ of 4.18 mg/kg body weight. This suppression was comparable with that of AS (98.7%) with an ED_50_ of 2.2 mg/kg body weight and CQ (90.5%) with an ED_50_ of 4.18 mg/kg body weight (Table 5). These drug combinations showed better inhibitory activity against *P. berghei in vivo* as was with *P. falciparum in vitro* with parasite suppression by the two drug combinations statistically significant (*p* < 0.05). A combination of AS and ANI provided better parasite inhibition both at ED_50_ drug combinations as well as at lower combination dosages. An ED_50_ drug combination between AS and ANI was able to clear the entire parasite, while a combination of half their respective ED_50_ values inhibited 67.0% parasite growth which was higher than the 50% inhibitory effect produced by the respective drugs administered individually. A quarter ED_50_ combination had a 35.4% growth inhibition. A 2.4 mg/kg body weight dose of CQ and 4.2 mg/kg body weight dose of ANI produced a 50% growth inhibition of *P. berghei* in mice when individually administered. However, a combination of these drugs' ED_50_ values had a much higher growth inhibition of 81%. A combination of half and quarter of their respective ED_50_ values cleared 27.2% and 10.2% of the parasites, respectively. This shows a synergistic effect between the activity of CQ and ANI though at high levels of their respective ED_50_ values only a much lower combination less than their respective ED_50_ produced a much lower inhibitory effect.

The drug combination ratios needed to attain 50% parasite chemosuppression was 0.88 mg/kg of AS combined with 1.68 mg/kg of ANI or 1.78 mg/kg of CQ combined with 3.15 mg/kg of ANI assuming there exists a linear relationship between the percentage chemosuppression and the combination ratios. This shows that when used in combination, less of the drugs is needed to attain the same therapeutic effect. This reduction in the concentrations used in combinations is due to the differences in the drug targets on the parasite metabolic pathways as elucidated in Introduction. The capacity of the combination drug agent to target different functional molecules within the parasites retards its chances of developing resistance as well as reduces chances of toxicity to the host animal and production costs [25].

In conformity to the less-drug-usage-higher-therapeutic-effect-and-low-toxicity principle above, observation of the mice posttreatment showed that there were high survival rates when combination drugs were employed compared to when they were administered individually. Remarkable parasitaemia suppression by the drugs in combination relative to the effect of the drugs individually administered was a classic example of drug synergy.

## 5. Conclusion

In principle, this study has demonstrated that ANI has antiplasmodial activity against 4-aminoquinoline-sensitive and -resistant *P. falciparum* lines. ANI combinations with either artesunate or chloroquine are more effective against *Plasmodia* with increased parasite clearance due to dual targets against the parasites. Potent antimalarial activity of ANI combinations with either AS or CQ implies that the combinations can be explored for future development of affordable but more effective antimalarial drugs that will mitigate growing resistance if their clinical efficacies can be demonstrated. The study recommends *in vivo* assays of the drug combinations in primate models towards clinical trials with human subjects.

## Figures and Tables

**Table 1:** *In vivo* individual drug assays.

Drug dosages	Animal test population groups			
	I	II	III	IV
AS (mg/kg/day)	5	2.5	1.25	0.65
CQ (mg/kg/day)	5	2.5	1.25	0.65
ANI (mg/kg/day)	10	5	2.5	1.25
Control	—	—	—	—

The starting concentrations were chosen based on the existing literature except for the novel drug ANI that was on trial.

**Table 2:** *In vitro* IC_50_ values of AS, CQ, and ANI against *Plasmodium* isolates.

Drug	CQ sensitive (3D_7_)	CQ resistant (W_2_)
	IC_50_ ± SD	IC_50_ ± SD
ANI	7.12 ± 0.17 *μ*g/ml	8.75 ± 0.3 *μ*g/ml
CQ	10.9 ± 1.1 ng/ml	48 ± 0.07 ng/ml
AS	1.6 ± 0.05 ng/ml	2.4 ± 0.1 ng/ml

ANI had lower *in vitro* antiplasmodial activity compared to AS and CQ with its values in *μ*g/ml.

**Table 3:** *In vitro* IC_50_ values for ANI in combination with either AS or CQ against *Plasmodium* isolates.

Combination	Strain	Combination quantities	FIC; AS or CQ	FIC; ANI	Sum FIC
AS/ANI	3D_7_	0.8 ng/ml AS + 2.6 *μ*g/ml ANI	0.5	0.36	0.86
	W_2_	1.1 ng/ml AS + 3.3 *μ*g/ml ANI	0.45	0.37	0.82
CQ/ANI	3D_7_	4.6 ng/ml CQ + 3.1 *μ*g/ml ANI	0.42	0.43	0.85
	W_2_	22 ng/ml CQ + 3.7 *μ*g/ml ANI	0.45	0.42	0.87

The drug combination concentrations were based on concentrations inhibiting 50% growth of parasite. With sum FIC values <1, these combinations are synergistic against the parasite.

**Table 4:** IC_50_ and selective index values for drug combinations against Vero E6 and 3D_7_ cells.

Drug	IC_50_ *μ*g/ml		
	Vero E6	3D_7_	SI
AS-ANI	35.43	2.6	13.6
CQ-ANI	41.13	3.1	13.3

Selective index for safety analysis: selective indices were over 10 times the ED_50_ values of individual drugs thus safe.

**Table 5:** Antiplasmodial activity of AS, CQ, and ANI on day 4 pi against *P. berghei* ANKA.

Drug	Inhibitory concentrations (mg/kg/day) on day 4 pi	
	ED_50_	ED_90_
AS	2.22	4.2
CQ	2.38	4.75
ANI	4.18	9.1

The effective doses inhibit growth of parasites by 50% (ED_50_). AS is two-fold more effective than ANI.

## Data Availability

The data used to support the findings of this study are included within the article. Any additional data are available from the corresponding author upon request.

## Abbreviations

AS: Artesunate
CQ: Chloroquine
ANI: 3-Chloro-4-(4-chlorophenoxy) aniline
IC50: Inhibitory concentration that reduces parasite growth by 50%
ED50: Effective dose that reduces parasitaemia by 50%
FIC: Fractional inhibitory concentration
WHO: World Health Organization
IRS: Indoor residual spraying
ITNs: Insecticide-treated nets
ACT: Artemisinin-based combination therapy
ACP: Acyl carrier protein
FASII: Fatty acid biosynthesis type II
KEMRI: Kenya Medical Research Institute
RBCs: Red blood cells
pi: Postinfection
ip: Intraperitoneally
OD: Optical density
CC50: Concentration causing 50% visible alteration of intact cells
CPM: Counts per minute.
